# The complete chloroplast genome of *Fraxinus hupehensis* and phylogenic analysis of Lamiales

**DOI:** 10.1080/23802359.2020.1829128

**Published:** 2020-10-12

**Authors:** Weirui Zhang, Peng Liu, Jiahui Liu, Yanxia He

**Affiliations:** a Eucommia Ulmoides Cultivation and Utilization of Henan Engineering Laboratory, Kaifeng, China; b School of Life Sciences, Henan University, Kaifeng, China

**Keywords:** Chloroplast genome, *Fraxinus hupehensis*, phylogenetic tree

## Abstract

*Fraxinus hupehensis* is a national rare and Endangered tree in the Oleaceae family, that has high commercial value owing to its slow growth, interlaced roots, intricate tree shape, and easy to shape. Here, we determined the complete chloroplast (cp) genome sequence for *F. hupehensis* using genome skimming sequencing. The cp genome was 155,698 bp and consisted of a large single copy (LSC) region (86,498 bp), a small single copy (SSC) region (17,803 bp) and two inverted repeats (IRs) (25,694 bp). It encodes 131 genes, including 88 protein-coding genes, 8 rRNAs and 35 tRNAs. Phylogenetic analysis indicates that *F. hupehensis* was relatively closely related to *F. chinensis* compared to other species in the Oleaceae family.

*Fraxinus hupehensis* Chu, Shang et Su, a woody plant of the Oleaceae family, has been officially listed as a national rare and Endangered tree species in China (Song et al. 2019). The species is only distributed in the southern foot of the Dahong Mountain in Hubei province, China, and has high commercial value owing to its slow growth, interlaced roots, intricate tree shape, and easy to shape. Therefore, it needs to be studied in details and prioritized as a conservation target. Phylogenetic relationships of species in the Oleaceae family remain one of the most problematic topics in angiosperm systematics (Yuan et al. 2010). The emergence of next generation sequencing technology makes it possible to obtain a large number of genomic data quickly (He et al. 2017; Liu et al. 2017). In this study, the first complete chloroplast genome of *F. hupehensis* was sequenced using genome skimming data. The raw sequence data has been deposited into NCBI SRA with project accession of PRJNA657087, and the genome sequence was registered in GenBank with the accession number MT812688.

Leaves of *F. hupehensis* were sampled from the campus of Henan University, China (34°49′19.78″N, 114°18′51.26″E), its voucher specimen was deposited at the Herbarium of Henan University (Accession number: HENU20200523). Total genomic DNA was extracted with the SDS method and high-quality DNA was sheared to a size of 350 bp. The paired-end library was then sequenced using Illumina NovaSeq PE150 at Beijing Novogene Bioinformatics Technology Co., Ltd. The complete chloroplast genome was assembled using a CLC Genomic Workbench 9.5.2 (CLC Inc., Aarhus, Denmark), with *Fraxinus chinensis* (GenBank accession number: MK299391) as a reference. The plastome was annotated using Geneious R11 (Biomatters, Auckland, New Zealand) following the description provided in Liu et al. (2018).

The plastome of *F. hupehensis* possessed a typical quadripartite structure with a length of 1,55,698 bp, containing two inverted repeat (IR) regions of 25,694 bp, a large single-copy (LSC) region of 86,498 bp, and a small single-copy (SSC) region of 17,803 bp. The new sequence comprised a total of 131 genes: 88 protein-coding genes, 8 ribosomal RNA genes, and 35 tRNA genes. Among these genes, nine protein-coding genes (*atpF, ndhA,rpoc1, ndhB, petD, rpl16, rpl2, rps12, and rps16)* contained one intron, whereas two genes (*clpP and ycf3*) contained two introns. The *rps12* gene was trans-spliced with the 5′ end located in the LSC and the 3′ end duplicated in the IR regions. The overall percentage of GC content was 37.8 and the corresponding values of the LSC, SSC, and IR regions were 35.9, 32.0, and 43.2, respectively.

The phylogenetic tree, including *F. hupehensis* and 18 other species inLamiales, was constructed based on whole plastome sequences using the maximum likelihood (ML) method performed using RAxML-HPC XSEDE v.8.2.8 from CIPRES (http://www.phylo.org/) (Stamatakis 2014), with *Capsicum annuum* used as the outgroup. The constructed tree revealed that *F. hupehensis* was relatively closely related to *F. chinensis* compared to other species in the Oleaceae family with a 100% bootstrap value (Figure 1). This result will provide valuable insight into conservation and evolutionary histories for this rare but important species.

**Figure 1.**
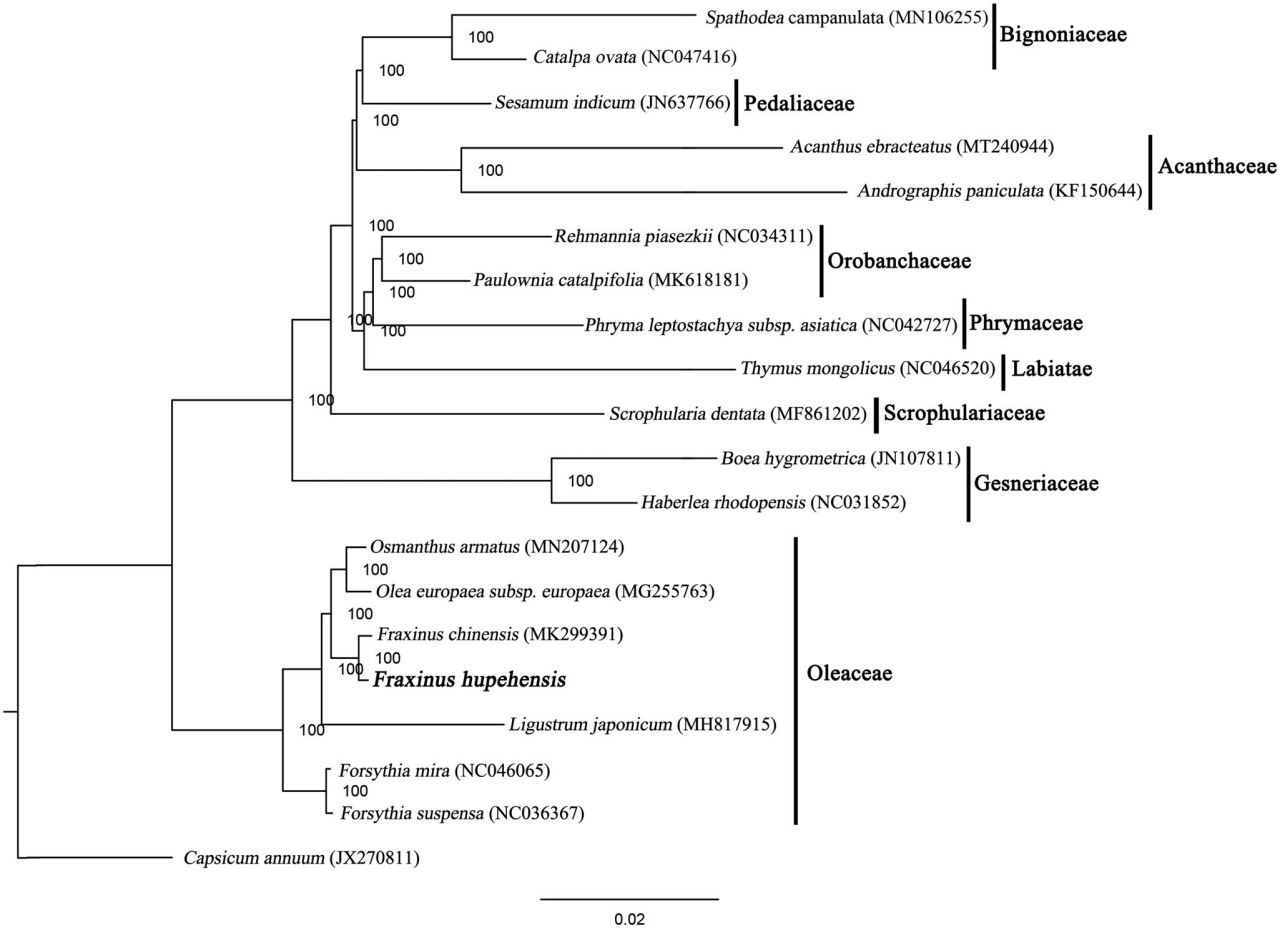
Phylogenetic relationships of Lamiales inferred based on whole chloroplast genome sequences. Number above each node indicates the ML bootstrap support values.

## Data Availability

The data that support the findings of this study are openly available in GenBank of NCBI at https://www.ncbi.nlm.nih.gov, reference number MT812688.
